# Clinical spectrum of myelin oligodendrocyte glycoprotein antibody-associated disease in Brazil: a single-center experience

**DOI:** 10.1055/s-0043-1777002

**Published:** 2023-11-30

**Authors:** Katharina Messias, Renata Moreto, Camila Aquino Cruz, Nathalia Rossoni Ronchi, Antonio Carlos dos Santos, André Messias, Vanessa Daccach Marques

**Affiliations:** 1 Universidade de São Paulo, Faculdade de Medicina de Ribeirão Preto, Departamento de Neurociências e Ciências do Comportamento, Ribeirão Preto SP, Brazil.; 2 Universidade de São Paulo, Faculdade de Medicina de Ribeirão Preto, Departamento de Oftalmologia, Otorrinolaringologia e Cirurgia de Cabeça e Pescoço, São Paulo SP, Brazil.; 3 Universidade de São Paulo, Faculdade de Medicina de Ribeirão Preto, Departamento de Imagens Médicas, Hematologia e Oncologia Clínica, Ribeirão Preto SP, Brazil.

**Keywords:** Myelin-Oligodendrocyte Glycoprotein, Optic Neuritis, Encephalomyelitis, Acute Disseminated, Myelitis, Magnetic Resonance Imaging, Glicoproteína Mielina-Oligodendrócito, Neurite Óptica, Encefalomielite Aguda Disseminada, Mielite, Imageamento por Ressonância Magnética

## Abstract

**Background** Anti-myelin oligodendrocyte glycoprotein (anti-MOG) antibody-associated disease (MOGAD) is an immune-mediated neurological disorder with a broad spectrum of clinical presentation that is often difficult to distinguish from other demyelinating diseases, such as multiple sclerosis and neuromyelitis optica spectrum disorder.

**Objective** To describe the clinical and paraclinical characteristics of MOGAD in a Brazilian tertiary center.

**Methods** We retrospectively reviewed the records of adult and pediatric patients who tested positive for anti-MOG antibodies and presented with clinical and radiological diseases compatible with MOGAD.

**Results** Forty-one patients (10 children) were included: 56% female, 58% Caucasian, mean age at onset 31 years (range 6-64), with a mean disease duration of 59.6 months (range 1-264 months). The most frequent onset presentation was optic neuritis (68%), acute disseminated encephalomyelitis (ADEM, 12%), and myelitis (10%). A monophasic disease course was observed in 49%. EDSS median was 2.1 at the last visit. Most patients (83%) were under continuous immunosuppressive treatment. Azathioprine was the first-line treatment in 59%. In all ADEM cases, conus, and root involvement was radiologically observed on MRI.

**Conclusion** Brazilian MOGAD patients presented with a similar spectrum of previously reported MOGAD phenotypes. Conus and spinal root involvement seems to be frequently present in MOGAD-ADEM and could serve as radiologic characteristics of this clinical entity.

## Resumo

**Antecedentes** A doença associada ao anticorpo da glicoproteína da mielina de oligodendrócitos (anti-MOG; MOGAD) é uma doença neurológica imunomediada com um amplo espectro de apresentações clínicas que muitas vezes é difícil de distinguir de outras doenças desmielinizantes, como a esclerose múltipla e o distúrbio do espectro da neuromielite óptica.

**Objetivo** Descrever as características clínicas e paraclínicas da MOGAD em um centro terciário brasileiro.

**Métodos** Revisamos retrospectivamente os prontuários dos pacientes adultos e pediátricos que testaram positivos para anticorpos anti-MOG e apresentaram um quadro clínico e radiológico compatível com MOGAD.

**Resultados** Quarenta e um pacientes (10 crianças) foram incluídos: 56% do sexo feminino, 58% caucasianos, idade média de início da doença foi 31 anos (intervalo de 6-64), com duração média da doença de 59,6 meses (intervalo de 1-264 meses). A apresentação inicial mais frequente foi neurite óptica (68%), seguida pela encefalomielite disseminada aguda (ADEM, 12%) e mielite (10%). Um curso monofásico da doença foi observado em 49%. EDSS foi de 2,1 na última visita. A maioria dos pacientes (83%) estava sob tratamento imunossupressor contínuo. Azatioprina foi o tratamento de primeira linha em 59%. Em todos os casos de ADEM, o envolvimento do cone medular e das raízes espinhais foi observado radiologicamente na ressonância magnética.

**Conclusão** Os pacientes brasileiros com MOGAD apresentam um espectro clínico e radiológico semelhante aos fenótipos de MOGAD relatados anteriormente. O envolvimento do cone e das raízes espinhais parece estar frequentemente presente no MOGAD-ADEM e poderia servir como característica radiológica nesta entidade.

## INTRODUCTION

Myelin oligodendrocyte glycoprotein (MOG) antibody-associated disease (MOGAD) is a demyelinating central nervous system (CNS) disorder that is associated with a serological antibody directed against MOG, a glycoprotein located on the myelin surface and found exclusively in the CNS. 1

The main clinical spectrum comprises optic neuritis (ON), myelitis, acute demyelinated encephalomyelitis (ADEM), cortical encephalitis, brainstem syndromes, and FLAIR-hyperintense lesions in anti-MOG-associated encephalitis with seizure (FLAMES). 2

Diagnostic criteria for MOGAD were recently published by an international MOGAD panel. 3 These emphasized the importance of using cell-based assays (CBAs) when testing for anti-MOG to avoid false positive results. Since August 2019, we have been performing anti-MOG testing in our facility using a commercially available CBA (Euroimmun AG, Lübeck, Germany).

Until now, few reports on MOGAD in the Latin-American population have been published, mainly due to difficulties in having access to anti-MOG testing. The Brazilian population presents an interesting environment because of its racial mingling. There are still many open questions concerning MOGAD, such as sex and/or racial predilection, recurrence rate, acute phase, and continuous treatment options, only to mention a few. Therefore, bringing together clinical and paraclinical presentations of MOGAD in different populations is of uttermost importance for understanding and better treating this relatively new and rapidly expanding neurological entity.

In the following article, we present the clinical and paraclinical characteristics, as well as the clinical outcome and disease course, of 41 patients who tested positive for anti-MOG antibodies in a tertiary hospital center in Brazil.

## METHODS

This was a retrospective descriptive study and it included all adult and pediatric patients who tested positive for anti-MOG in our facility: Hospital das Clínicas, Ribeirão Preto, University of São Paulo, Brazil. The study was approved by our hospital's ethics committee board and written informed consent was obtained from all patients or their legal representatives. All medical charts of individuals who tested seropositive for anti-MOG antibodies between August 2019 and April 2023 were reviewed. Patients who were lost on follow-up were contacted and all but one patient (a girl with multiphasic ADEM) returned for ambulatory evaluation.

The inclusion criteria consisted of a serologically positive anti-MOG test, using a commercially available fixed cell-based assay with native MOG as the substrate (Euroimmun AG, Lübeck, Germany), as well as presentation of typical clinical-radiological manifestations of MOGAD. All patients tested negative for AQP4-IgG using CBA (Euroimmun AG, Lübeck, Germany), and none of the patients fulfilled the 2017 McDonald criteria for multiple sclerosis. 4 Following the manufacturer's recommendation, an assay dilution of 1:10 was used for anti-MOG and anti-AQP4 testing, and visual observation was performed by an experienced laboratory assistant. All patients fulfilled MOGAD diagnostic criteria. 3

The following data were obtained: age at onset, sex, race, first clinical presentation, vaccination or recent infection, number of relapses, type of relapse, disease course and duration, Expanded Disability Status Scale (EDSS) during first attack (nadir) and at last follow-up visit, cerebrospinal fluid (CSF) findings, presence of antinuclear antibody (ANA), concurrent autoimmune disease, and whether acute and/or continuous treatment were prescribed. Neurological involvement was categorized into optic neuritis (ON), ON plus myelitis (ON + MY), myelitis (MY), ADEM, or FLAMES/cortical encephalitis. A relapse was defined as any new CNS symptom/sign lasting more than 24h in the absence of other causes, and occurring more than 30 days after the previous attack.

Brain and spinal cord MRI findings were included if performed during an acute attack of first clinical presentation. MRI scans were performed with 1.5T or 3T scanners. According to institutional protocol brain MRI included sagittal and axial 3D T1-weighted image (T1WI), axial turbo spin-echo T2-weighted image (T2WI), axial/sagittal 3DFLAIR and post-contrast axial T1WI image. In cases of suspected optic neuritis, brain MRI was extended by axial and coronal T2WI with fat saturation and fat-saturated post-contrast axial and coronal images for better orbital evaluation. The spinal protocol included sagittal T1, T2, and axial T1 proton-density WIs, followed by sagittal and axial T1WI after gadolinium administration.

Categorical variables are presented as frequencies and percentages, while continuous variables are presented as medians or means and ranges. Averages were presented as percentages.

## RESULTS

### Demographics

Forty-one patients met the inclusion criteria and were enrolled. Clinical and paraclinical data are summarized in Table 1 . A slight majority were females (n = 23; 56.1%), with a strong female predominance in the pediatric cohort (80%). The mean age of the cohort was 35.8 years, while the mean age at disease onset was 31.0 years (range 6-64). Twenty-four patients (58.8%) were Caucasians.

**Table 1:** Summary of the epidemiologic, clinical and laboratory characteristics of the MOGAD cohort

Variable	All patients (n = 41)	Pediatric onset (n = 10)	Adult-onset (n = 31)
Female, % (n)	56.1 (23)	80.0 (8)	45.0 (14)
Caucasian/white, % (n)	58.8 (24)	70.0 (7)	54.8 (17)
Mean age, years (range)	35.8 (8-71)	14.3 (8-36)	42.7 (23-71)
Age at disease onset, mean years (range)	31.0 (6-64)	10 (6-15)	37.8 (18-62)
Disease duration, mean months (range)	59.6 (1-264)	55 (1-256)	61.2 (2-264)
**Symptoms at disease onset**	% of patients (n)		
Optic neuritis	68.3 (28)	30 (3)	80.1 (25)
Optic neuritis + concomitant myelitis	4.9 (2)	0	6.5 (2)
ADEM (+/− optic neuritis and/or myelitis)	12.2 (5)	50 (5)	0
Myelitis	9.8 (4)	10 (1)	9.7 (3)
Others: FLAMES; cortical encephalitis	4.9 (2)	10 (1)	2.4 (1)
Infectious prodrome/vaccination before disease onset, % (n)	34 (14)	60 (6)	25.8 (8)
EDSS during first attack at nadir, average (range)	4.4 (1-8.5)	6.1 (3-8.5)	2.5 (1-8.5)
EDSS at final visit, average (range)	1.9 (0-8)	1.0 (1-4)	2.25 (0-8)
**Relapses**	% of patients (n)		
Relapsing disease course	51.2 (21)	60 (6)	48.4 (15)
Number of relapses per patient (range)	2.5 (1-13)	2 (1-3)	2.6 (1-13)
Number of relapses	n = 53	n = 8	n = 45
Optic neuritis	69.8 (37)	50 (4)	73.3 (33)
Optic neuritis + myelitis	13.2 (7)	25 (2)	11.1 (5)
Myelitis	13.2 (7)	0	15.6 (7)
ADEM	1.9 (1)	1 (12.5)	0
Optic neuritis at any time point, % (n)	82.9 (34)	60 (6)	90 (28)
Other Autoimmune disease	2.4 (1)	0	3.2 (1)
**Laboratory**			
Positive antinuclear antibodies, n = 36	7.3 (3)	0	9.6 (3)
Mean CSF leucoyctes (cells/mm ^3^ ), range	22.3 (0-200)	67.9 (2.7-200)	8.4 (0-80)
> 5 leucocytes/mm ^3^ , % (n)	43.9 (18)	70 (7)	32.3 (10)
Mean CSF protein (mg/dL), range	36.9 (12-70)	41.1 (30-70)	34.6 (12-69)
> 45 mg/dL protein % (n)	26.8 (11)	30 (3)	25.8 (8)
Positive oligoclonal bands, n = 29	17.2 (5)	12.5 (1 out of 8)	19.0 (4 out of 21)
**Treatment**	% of patients (n)		
Acute treatment at onset	80.5 (33)	90 (9)	77.4 (24)
• IVMP	45.4 (15)	20 (2)	54.2 (13)
• IVMP + PLEX	36.4 (12)	30 (3)	37.5 (9)
• Oral or intramuscular steroids	9.1 (3)	10 (1)	8.3 (2)
• IVIG	9.1 (3)	30 (3)	0
• Oral steroid taper	46.3 (19)	60 (6)	41.9 (13)
Chronic treatment ever used	82.9 (34)	70 (7)	87.1 (27)
** 1 ^st^ line chronic treatment **	% of patients (n)		
• Azathioprine	58.8 (20)	57.1 (4)	59.3 (16)
• Rituximab	32.4 (11)	28.6 (2)	33.3 (9)
• Other	8.8 (3)	14.3 (1)	7.4 (2)
Treatment change	38.2 (13)	1 (14.3)	44.4 (12)

Abbreviations: ADEM, acute disseminated encephalomyelitis; CSF, cerebrospinal fluid; EDSS, expanded disability status scale; FLAMES, FLAIR-hyperintense lesions in anti-MOG-associated encephalitis with seizure; IVMP, intravenous methylprednisolone; PLEX, plasma exchange. Note: Addendum: Only 36 patients were tested for ANA.

### Clinical presentation

The most frequent initial presentation was ON (28 patients; 68.3%), with bilateral ON in 32.1%. In 17 cases, fundoscopy was registered during acute phase, and optic disc edema was present in 10 patients (58.8%). Headache associated with ON was described in 21 patients (75%). The second and third most frequent phenotype at onset was ADEM (5 pediatric patients, 12.5%) and transverse myelitis (4 patients, 9.8%). Simultaneous ON associated with myelitis and FLAMES/cortical encephalitis were present in 2 patients (4.9%), respectively. In the pediatric subgroup 5 children (50%) presented at onset with ADEM, 3 (30%) with ON, one (10%) with myelitis, and one (10%) with cortical encephalitis associated with myelitis ( FIgure 2 ).

An infectious prodrome or vaccination within the 30 days preceding MOGAD onset was reported in 14 cases (34%). In the pediatric subgroup infectious prodrome or vaccination was present in 60% and in all cases of ADEM.

The median disease duration was 59.6 months (range 1-264). Twenty-one patients (51.8%) presented with relapsing disease: ON was the most frequent relapse type (37 relapses, 75%); followed by ON combined with myelitis or myelitis alone in 7 cases (13.2%), respectively. One child presented with multiphasic ADEM. The median number of relapses per patient was 2.5 (range 1-13), and 14 patients (34.1%) presented 3 or more relapses. Taking into account all relapses, 32 patients (82.9%) presented with ON at some point.

**Figure 1:**
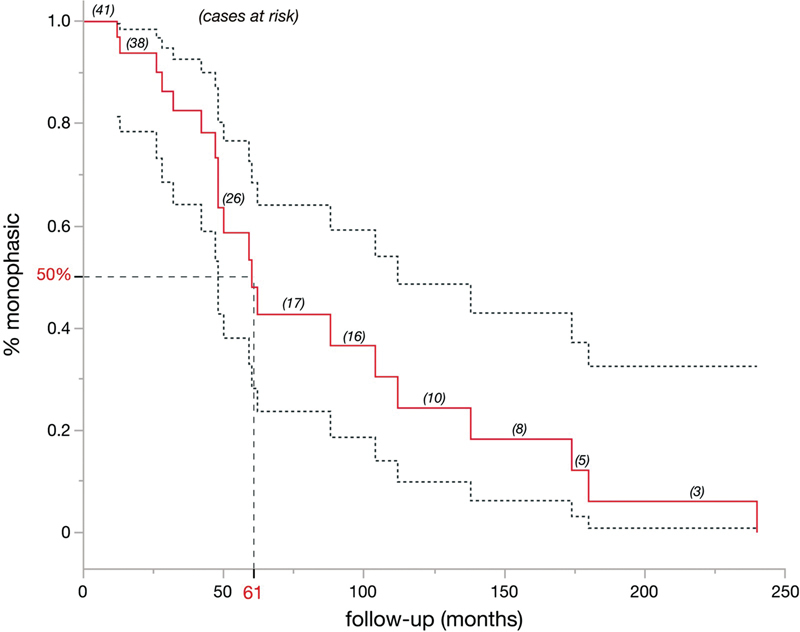
The red line represents the Kaplan-Meier survival analysis calculated for the risk of a relapsing disease course for the total cohort over the follow-up period. Dashed lines indicate the 95% confidence limits. All 41 patients initially showed only one disease manifestation (monophasic), and the number of patients at risk for relapse is provided for various time points of interest (within parenthesis). In addition figure highlights that 50% of patients with disease duration longer than 61 months showed a relapsing course.

Among the patients with a disease duration of 60 months or more (n = 13), only 1 patient (7.8%) remained relapse-free. Among those with a disease duration of 24 months or less (n = 14), the majority (78.6%; 11 patients) did not have a relapse. The Kaplan-Meier curve for relapse-free survival based on the time of disease onset is shown in Figure 1 . To overcome the bias in superestimating relapsing course in our MOGAD cohort simply by the fact that MOGAD may be less frequently diagnosed in monophasic presentation, we looked at 22 patients that were diagnosed with MOGAD after the first manifestation. Of these only 2 patients relapsed, however, only 5 patients were under no continuous immunosuppressive treatment.

**Figure 2:**
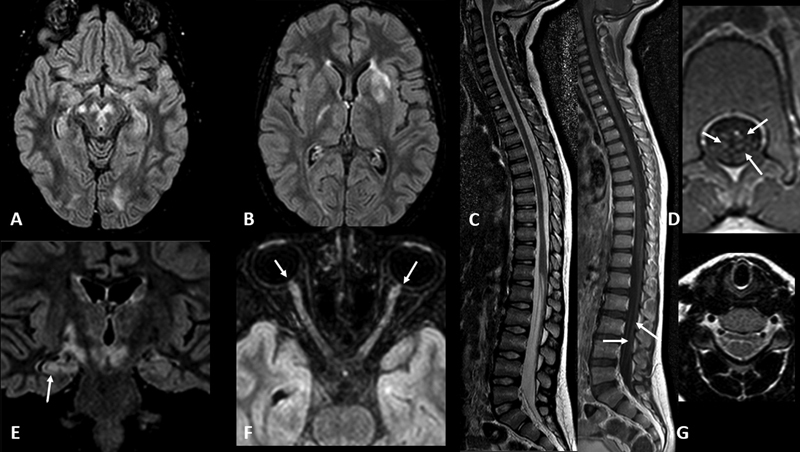
MRI findings of 5-year-old female patient with optic neuritis, combined central and peripheral demyelination, and peripheral enhancement of the ventral and dorsal nerve roots of the cauda equina. All the imagens are from the same date. Axial FLAIR with fat suppression (A,B, and F), coronal FLAIR (E), sagittal TSE T2 and T1 after gadolinium administration (C), axial T1 (D), and axial T2-weight image (G). The arrow points at contrast enhancement of the cauda equina (D), presence of hippocampal, corticospinal tract and mesencephalic lesions (E), and bilateral and symmetrical optic nerve head swelling (F).

The median EDSS was 4.4 (range 1-8.5) during the nadir of the first relapse, and 2.1 (range 0-8) at the final visit.

One patient (2.4%) presented with concomitant autoimmune disease (primary hypothyroidism). Antinuclear autoantibodies were positive in three patients (7.3%).

Comparing clinical presentation found for Caucasians and non-Caucasian individuals, there were no significant differences regarding initial presentation, clinical onset phenotype, age at onset, final EDSS score, and number of relapses.

### Laboratory testing

MOG testing was performed in 26 patients (63.4%) during the acute disease attacks. The median number of relapses that occurred before MOG testing was performed was 2.3 (range 1-9). CSF results were available for all but two patients. The white blood cell count ranged from 0-200 cells/mm ^3^ (mean of 22.3 cells/mm ^3^ ). Eighteen patients (43.9%) presented with 5 or more white blood cells/mm ^3^ . The mean CSF protein concentration was 36.9 mg/dL (range 12-70), and 11 patients (26.8%) had elevated protein levels of more than 45 mg/dL. In 29 patients, oligoclonal bands were investigated, and 5 patients (17.2%) were found to be positive.

### MRI characteristics at disease onset

Twenty-five MRI scans performed at disease onset were available (61%). The most frequent finding was ON (48%, n = 12), mostly unilateral (67%, n = 8). In 75% (n = 9) more than 50% of the optic nerve was radiologically involved. Chiasmal involvement was present in one patient (8%). Perineuritis and contrast enhancement were observed in 10 patients (83%), respectively. The second most common MRI phenotype was myelitis (24%, n = 6). Usually, myelitis was longitudinally extensive (83%, n = 5) with central gray matter involvement (83%, n = 5). Contrast enhancement was present in 67% (n = 4) and conus involvement was detected in 33% (n = 2). ADEM was the third most frequent MRI pattern (20%, n = 5). In all patients with ADEM spinal MRI was performed, and all patients had radiological conus and spinal root involvement. Radiological findings are summarized in Table 2 .

**Table 2:** Magnetic resonance findings at disease onset

**MR findings available at disease onset (n = 25/41), 61%**			
Optic neuritis	48% (n = 12)	Unilateral, % (n)	67 (8)
		Length > 50%	75 (9)
		Anterior involvement	92 (11)
		Chiasmal involvement	8 (1)
		Perineuritis	83 (10)
		Contrast enhancement	83 (10)
		Brain lesions	17 (2)
Myelitis	24% (n = 6)	LETM, % (n)	83 (5)
		Central GM involvement	83 (5)
		Conus involvement	33 (2)
		Contrast enhancement	67 (4)
ADEM	20% (n = 5)	Brain lesions only, % (n)	0 (0)
		Brain lesions + myelitis	60 (3)
		Brain lesions + ON	0 (0)
		Brain lesions + myelitis +ON	40 (2)
		Conus and root involvement	100 (5)
FLAMES/cortical encephalitis	8% (n = 2)	Brain lesions only, % (n)	50 (1)
		Brain lesions + myelitis	0 (0)
		Brain lesions + ON	50 (1)

Abbreviations: ADEM, acute disseminated encephalomyelitis; FLAMES, FLAIR-hyperintense lesions in anti-MOG-associated encephalitis with seizure; GM, grey matter; LETM, longitudinally extensive transverse myelitis; ON, optic neuritis.

### Treatment

Thirty-three patients (80.5%) received acute treatment during the first attack. Fifteen patients (45.4%) received intravenous methylprednisolone (IVMP), 1000 mg for 3-5 days. Twelve patients (36.4%) received IVMP followed by plasma exchange. Three patients (9.1%) received oral or intramuscular steroids, and three (9.1%) patients received intravenous immunoglobulin (IVIG). Maintenance immunosuppression was prescribed in 34 patients (82.9%). Nearly half of the subjects received an oral steroid taper, however, treatment duration and dosing were highly heterogeneous among the group (1-12 months of oral steroid treatment, usually 1 mg/kg/d in the first 3 months, reduction to 10-20 mg/d afterward and maintenance for usually 6 months). The mean number of relapses before continuous treatment was started was 2.15. As first-line therapy, 20 patients (58.8%) received azathioprine, 11 rituximab (32.4%), and 3 other medications. Sixteen patients (39.0%) started immunosuppressive maintenance therapy after their first attack, 11 patients (26.8%) after 2 attacks, and 7 patients (17.1%) after 3 or more attacks.

Thirteen patients (38.2%) required treatment alterations due to failure or adverse effects. Rituximab was the most often prescribed second-line treatment (n = 6; 46.2%), followed by azathioprine and immunoglobulin (n = 2, each; 23.1%).

## DISCUSSION

In this study, we describe the clinical data of 41 pediatric and adult cases with a diagnosis of MOGAD that were cared for in a tertiary Brazilian center. Our cohort presented a slight female predominance of 56.1%. This contrasts with older data, which demonstrated higher female proportions of 60-70% in different populations: Chile, 5 USA, 6 7 Europe, 8 China 9 and UK. 10 On the other hand, a similar sex distribution of 1:1 was detected in French 11 and Dutch 12 cohorts. Previous data from a small Brazilian/Japanese cohort of 16 anti-MOG-positive patients showed a slight male predominance of 62.5%. 13 There certainly still remains some doubt about the precise sex distribution of MOGAD, but the strong female predominance of nearly 90% that has been observed in relation to NMOSD 14 has not been found in MOGAD. Interestingly, the pediatric cohort presented with a high female prevalence (80%).

The predominance of Caucasians (61.1%) was also in line with findings from other studies. 6 7 12 However, it is important to state at this point that the Brazilian population has a high degree of miscegenation. Therefore, it is difficult to determine who is considered “Caucasian”. A study conducted at a neurological center in Rio de Janeiro, Brazil, on a population predominantly of African descendants, detected a low prevalence of anti-MOG in a cohort of patients diagnosed with NMOSD-anti-aquaporin-4 seronegative demyelinating CNS disease. 15 Another Brazilian group investigated MOGAD prevalence in a tertiary center located in São Paulo, and confirmed that there was a lower prevalence of MOGAD, compared with MS and NMOSD. 16 Therefore, genetic and environmental influence seems possible in MOGAD, as already known for NMOSD, which has a higher prevalence among East Asians and blacks than among whites. 14 17 In our cohort, we found no differences regarding clinical presentation between Caucasians and non-Caucasians, however, further population-based studies on MOGAD are needed in order to address this matter.

Age at symptom onset showed a wide range (6-64 years) with a mean age at onset of 35.8 years, which is concordant with previously presented data. 5 8 10 As also described in previous reports, the most frequent pediatric phenotype at onset was ADEM (50%), followed by ON (20%). 18 In the adult population, ON was present in 83.4% at onset (in a third bilateral) followed by myelitis in 9.7%, which is also in line with previously published data. 6 8 11 It is noteworthy that optic disc swelling was observed in 58.8% of all ON cases in which fundus examination data were available. This once again emphasizes the predilection of involvement of anterior parts of the optic nerve in MOGAD, 19 a clinical feature usually not observed at such high frequency in MS 20 or NMOSD. 21 Furthermore, severe headache was reported in 75% of the ON cases and had already been described in prior reports on MOGAD. 22 23 24 Oftentimes headache preceded the onset of visual loss by some days or even weeks, was reported as intense and migraine-like, and usually responded well to steroid treatment.

**Figure 3:**
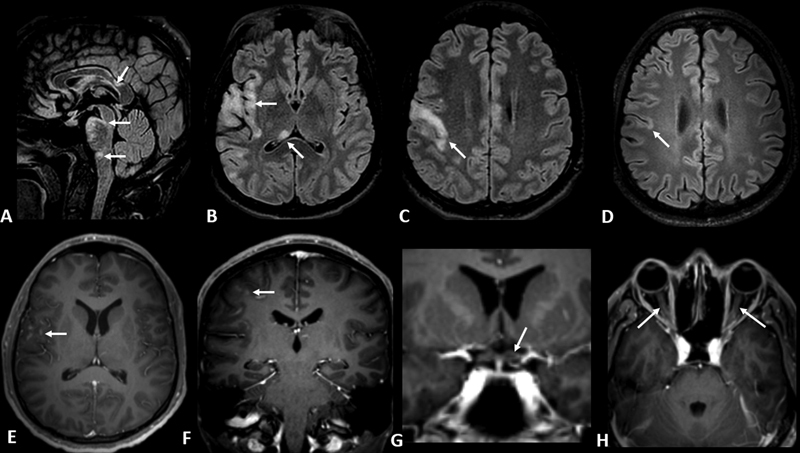
MRI findings of patient diagnosed with FLAMES (FLAIR-hyperintense lesions in anti-MOG-associated encephalitis with seizure). A 23-year-old previously healthy man developed severe headache, left sided hemiparesis, bilateral blurry vision, mental confusion and first epileptic seizure 3 weeks after receiving the Pfizer-BioNTech COVID-19 mRNA vaccine. Brain MRI images before steroid pulse therapy are shown: 3D-FLAIR with fat suppression (A-D); 3DT1 with fat suppression and after gadolinium administration (E-H). The arrows indicate lesions in the corpus callosum, brainstem, and cortex. Lesions are observed with perivascular CLIPPERS (chronic lymphocytic inflammation with pontine perivascular enhancement responsive to steroids) in image A, and cortical lesions with contrast enhancement in images E and F. There is peripheral enhancement of the chiasm (G) and bilateral optic nerves (H).

Infectious prodrome or vaccination within the last 30 days preceding disease was reported in about a third of patients. MOGAD after SARS-CoV-2 infection has already been described, 25 26 27 28 as well as MOGAD after COVID vaccination. 29 30 31 Two of our patients presented with MOGAD after COVID vaccination: a 22-year-old male, developed FLAMES about 3 weeks after receiving the Pfizer-BioNTech COVID-19 vaccination ( Figure 3 ); and a 43-year-old woman presented with transverse myelitis two weeks after receiving the Sinovac-CoronaVac vaccine. To our knowledge, this is the first reported MOGAD case after this type of vaccine.

In relation to radiological findings, the presence of cauda equina and spinal nerve root involvement in all ADEM cases was remarkable. An overlapping central and peripheral nervous system involvement with combined central and peripheral demyelination was already observed in MOGAD by other authors 32 and still is poorly understood. The authors hypothesized a “spreading” autoimmunity as a possible mechanism, among others: in this scenario, the high inflammatory state in ADEM with a presumed presence of soluble MOG in CSF could possibly trigger an autoimmune mimicry with peripheral myelin proteins. Cauda equina and spinal root involvement in ADEM-MOGAD could possibly serve as a radiological feature and should be addressed in future studies.

Among the cases of relapses, the most frequent clinical phenotype was also ON both for children and adults, and 82.9% of all patients presented with ON at some point. The same findings were observed in previous studies. 6 8 In our cohort, a slight majority of the patients presented relapsing disease (51.2%). The Kaplan-Meier curve for relapse-free survival based on the time of onset is shown in Figure 1 and illustrates the growing chance of suffering relapse with longer follow-up, as was also previously described. 8 Among the patients with a disease duration of 60 months or more (n = 13), only 1 patient (7.8%) remained relapse-free in our cohort. One patient presented with an interval between disease onset and relapse of more than a decade: this emphasizes the difficulty in deciding which patients should be treated with continuous immunosuppressive therapy

In the acute stage, nearly all patients were treated with high-dose IVMP, plasma exchange, or both. In our service, we have a relatively high percentage of plasma exchange (36.4%), compared with other groups. This can be explained by the severity of cases transferred to our service and local treatment strategies. Nearly half of the subjects, and all of the ADEM cases, received oral steroid taper after acute treatment intervention. However, steroid treatment duration and dosing were highly heterogeneous in this group. In our hospital, we usually start with an immunosuppressive dose of 1 mg/kg/d for at least 1 month. After 1-3 months, we slowly reduce the dose to 10-20 mg/d, which we maintain for an additional 3-6 months. It seems that oral steroid taper reduces the risk of further relapses, for both pediatric 33 and adult patients, 34 yet, further data are needed to determine the exact dosing and timing of oral steroid taper.

Another important observation was the high proportion of patients under continuous immunosuppressive treatment (82.9%). In 41% of the patients, continuous treatment was started after disease onset, usually based on presentation of severe disease or important sequelae. Azathioprine was the first-line medication most often used. The justification for using azathioprine is its relatively low cost, compared with other immunosuppressive medications, along with its convenient oral route. The second most often used medication was rituximab. Mycophenolate mofetil or IVIG were not used in our cohort as the first-line drug, due to their unreliable distribution in our country and high cost. Our cohort was too small for a relapse rate for each single therapy to be precisely reported.

Despite the frequent attacks, the median EDSS at the final follow-up was 2.1. However, there was a broad range of 1-8. A similar EDSS of 1.5 was observed in other studies. 5 6 35 Interestingly, the pediatric subgroup presented with higher EDSS at nadir during the first clinical event, however, they showed a better recovery with lower EDSS at the final visit. Surely, the frequent ADEM phenotype with a severe disability but good recovery could explain this observation, such as the possible better neuroplasticity in pediatric MOGAD. 36

Much less frequently than in previous reports (with rates of 11% 11 and 21.7% 6 ), only one of our patients (2.4%) presented with another autoimmune disease (Hashimoto thyroiditis). Furthermore, only 7.3% of our cohort were positive for the presence of antinuclear antibodies (ANA), which was similar to data from a study in Baltimore, in which 4% were positive for ANA. 6 These results contrasted with the percentages from other cohorts: Chile (36% 5 ), Europe (42.2% 8 ) and France (20.1% 11 ). Oligoclonal bands were detected in five patients out of 29 tests (17.2%), similar to what had been found previously. 5 6

There are certainly several limitations to this study, above all its retrospective design and the low number of patients included. A possible bias of overestimating relapse risk by recruiting individuals referred to our center years after disease onset because of relapse cannot be denied. It would be of great interest to observe patients diagnosed with MOGAD after their first clinical manifestation in order to get a possibly more accurate relapse risk in MOGAD. Furthermore, there were the limitations of the fixed-CBA test used in our cohort and the low threshold of 1:10. However, all of our patients fulfilled the recently published MOGAD criteria. 3

In conclusion, we have described the Brazilian MOGAD cohort cared for in a single center. Many demographic and clinical characteristics had already been described and were also present in our cohort, reinforcing the presence of a common MOGAD phenotype in different populations. We confirmed that there was a high prevalence of optic neuritis associated with optic disc swelling and severe headaches. Relapsing disease course seems to be more frequent in patients with long follow-ups, however, prospective studies are needed to address this question. Conus and spinal root involvement seems to be frequently present in ADEM and could serve as radiologic characteristic of MOGAD-ADEM.
